# Toward Supplementation Guidelines for Vegan Complementary Feeding

**DOI:** 10.1002/fsn3.4565

**Published:** 2024-11-01

**Authors:** Christian Koeder

**Affiliations:** ^1^ Faculty of Medicine, Institute for Prevention and Cancer Epidemiology University of Freiburg Freiburg Germany

**Keywords:** iodine, nutrition, plant‐based diet, selenium, vegan, veganism, weaning

## Abstract

Previously published recommendations for vegetarian (including vegan) diets for children have highlighted the need for vitamin B12 supplementation. Increased attention to several other key nutrients (including iodine, vitamin D, calcium, and iron) has also been recommended. However, an overview focusing on supplementation guidelines, specifically for vegan infants, has not been published, and a potential requirement for iodine and/or selenium supplementation in (some) vegan infants has not been discussed. Vegan complementary feeding should be supplemented (particularly with 5 μg/day of vitamin B12 and 10 μg/day of vitamin D). Iodine should be supplemented (up to 110 μg/day) if the intake of breast milk and infant formula is low, and selenium supplementation (5 μg/day) should be considered in regions with low soil selenium levels. Caution is required to avoid excessive intakes of iodine and particularly selenium. Supplements for vegan infants are on the market, and observational studies are urgently needed to assess the nutrient intake (including supplements) and status in vegan infants.

## Graphical abstract

Infants of vegan mothers are often transitioned from exclusive breastfeeding to purely vegan complementary feeding. Vitamin B12, iodine, vitamin D, calcium, iron, zinc, and selenium are nutrients of potential concern for vegan infants. Guidelines for supplementation during vegan complementary feeding are required to ensure adequate nutrition in this population.

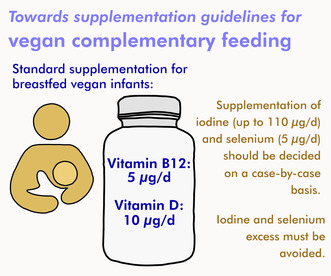

## 1 Objective

The objective of the present article is to provide suggestions for preliminary guidelines regarding nutrient supplementation during vegan complementary feeding, i.e., starting at an age of 4–6 months (Fewtrell et al. 2017; World Health Organization 2023) and continuing until the age of 1 year. It is the position of the present article that such supplementation guidelines are needed in order to prevent the occurrence of nutrient deficiencies and their consequences in this population group.

## 2 Introduction

Vegan and near‐vegan diets are becoming increasingly popular. Studies regarding the physical and mental development of vegan infants are still scarce, however. Popular sources of nutrition information (e.g., on video streaming platforms) are frequently not evidence‐based, and this also applies to sources of information about vegan nutrition. At the same time, the scarcity of studies in this field, particularly regarding infant nutrition, makes it challenging for scientists to formulate evidence‐based recommendations. Nevertheless, such information is urgently required, even if it must be extrapolated from existing (albeit limited) evidence about vegan nutrition in general (Koeder and Perez‐Cueto 2024), sparse existing evidence regarding vegan pregnancy and lactation (Pawlak et al. 2018; Perrin et al. 2023), as well as practical experience (suggestions for vegan infant feeding have been published since at least the late 1940s (Mayo 1948)).

It is known that without adequate supplementation, vegan diets may result in severe nutrient deficiencies, particularly vitamin B12 deficiency, whose consequences can be grave, irreversible, and even life‐threatening. Reports of severely deficient vegan infants in the medical literature refer to isolated cases, particularly of vitamin B12 deficiency, with a very small number of other (e.g., iodine or vitamin D) deficiencies reported. Less severe deficiency states might, however, still result in adverse effects on the cardiovascular system (Pawlak 2015) or bone health (Pawlak 2021), although this is not entirely certain.

While nutrition scientists may have a higher tolerance for remaining in the present state of uncertainty regarding a niche field such as vegan complementary feeding, it is those health professionals with direct contact to the general population, particularly dietitians, pediatricians, family physicians, and midwives, who urgently require guidelines on this niche topic. In the absence of evidence‐based, peer‐reviewed guidelines and appropriate guidance by health professionals, vegan parents are left to “do their own research,”, with a high likelihood of them finding predominantly non‐science‐based information (with video streaming and social media algorithms highlighting sensationalized content). While it would be unethical to hide the uncertainty associated with such preliminary guidelines, it would also be unethical to not attempt to formulate guidelines. To this effect, a draft for preliminary guidelines for the supplementation of vegan complementary feeding is given below. Food‐based recommendations for vegetarian (including vegan) complementary feeding have been published, including recommendations for vitamin B12, vitamin D, and EPA/DHA supplementation (Agnoli et al. 2017; Baroni, Goggi, and Battino 2019). The reader is also referred to general guidelines for complementary feeding (Fewtrell et al. 2017; World Health Organization 2023).

### 2.1 Key Nutrients

It can be proposed that there are 10 key nutrients (vitamin B12, calcium, vitamin D, iodine, omega‐3 fatty acids, iron, zinc, vitamin A, selenium, and protein) in the context of vegan diets in general (Koeder and Perez‐Cueto 2024) as well as in the case of vegan complementary feeding. Furthermore, attention to some of these nutrients should be prioritized, namely (first) vitamin B12, (second) iodine, and (third) vitamin D. Suggestions for both infant and maternal supplementation during complementary feeding are given in Table 1.

**TABLE 1:** Supplement recommendations for vegan infants during the first year of life.

Nutrient (infant age)	Infant			Maternal		Notes
	DRI range [UL range]	SPPL		DRI range [UL range]	SPPL	
Vitamin B12 (from 4 to 6 months)	0.4–1.5 μg/day (European Food Safety Authority 2017; Institute of Medicine 1998) [no UL (European Food Safety Authority 2024)]	5 μg/day (Agnoli et al. 2017; Baroni, Goggi, and Battino 2019; Lemale et al. 2019) (suggestion: no more than 10 μg/day [> 600% the DRI])		2.8–5 μg/day (European Food Safety Authority 2024; Institute of Medicine 1998) [no UL (European Food Safety Authority 2024)]	10–50 μg/day (Agnoli et al. 2017; Koeder and Perez‐Cueto 2024) (suggestion: no more than 200 μg/day [> 4000% the DRI])	There seems to be a consensus that all vegan infants who do not received infant formula should receive vitamin B12 SUPPL. Nevertheless, BM of adequately supplemented vegan mothers may be a sufficient source. To provide additional security, a daily supplement for the infant is recommended.
Iodine (from 4 to 6 months)	70–130 μg/day (European Food Safety Authority 2017; Institute of Medicine 2001) [UL from 1 year: 200 μg/day (Andersson and Braegger 2022; European Food Safety Authority 2024; Institute of Medicine 2001)]	BM consumed	SPPL	~200–290 μg/day (European Food Safety Authority 2017; Institute of Medicine 2001) [UL: 600–1100 μg/day (European Food Safety Authority 2024; Institute of Medicine 2001)]	~200–290 μg/day [100% DRI]. If iodized salt is used, the SPPL dose can be reduced accordingly.	Iodine SUPPL should be decided on a case‐by‐case basis. Excessive iodine intake must be avoided. Neither mother nor child should rely on seaweed (Andersson and Braegger 2022) as their (main) source of iodine. Salt should not be used for the infant's food (Baroni, Goggi, and Battino 2019; Fewtrell et al. 2017) although the WHO does not make a recommendation against salt in their complementary feeding guideline (World Health Organization 2023).
		~700–1000 mL/d	No SPPL [assuming a BMIC of 100–200 μg/L, an intake of 70–200 μg will be achieved from BM alone]			
		~500 mL/d	Up to 30 μg/day; no SPPL required [assuming a BMIC of 100–200 μg/L, an intake of ~50–100 μg will be achieved from BM alone]			
		< 500 mL/d	~30 μg/day [assuming a BMIC of 100–200 μg/L, an intake of < 80–130 μg will be achieved from BM + SPPL]			
		No or close to no BM (not recommended (Baroni, Goggi, and Battino 2019))	~70–110 μg/day [~100% of the DRIs](European Food Safety Authority 2014; Institute of Medicine 2001)			
Vitamin D (from the first few days of life)	10 μg/day (European Food Safety Authority 2017; Institute of Medicine 2011) [UL: 25–38 μg/day (European Food Safety Authority 2024; Institute of Medicine 2011)]	~10 μg/day (Baroni, Goggi, and Battino 2019) [100% DRI] (no more than 25–30 μg/day) unless there is a medical requirement for a higher dose (Institute of Medicine 2011)		15 μg/day (European Food Safety Authority 2017; Institute of Medicine 2011) [UL: 100 μg/day (European Food Safety Authority 2024; Institute of Medicine 2011)]	~15 μg/day [100% DRI] (unless there is sufficient endogenous synthesis from sunshine (European Food Safety Authority 2017))	There seems to be a consensus that all vegan infants should receive vitamin D SUPPL. Furthermore, SPPL is standard practice for all infants. Vitamin D2 is vegan. Vegan vitamin D3 is available. Benefits of vitamin D2 over D3 are uncertain.
Omega‐3 fatty acids (from 4 to 6 months)	ALA	0.5 E% (i.e., ~2.7–3.7 kcal/d or ~ 0.3–0.4 g/d) (European Food Safety Authority 2017) [no UL]	ALA‐rich foods should be given: Flaxseed oil (~1/4–1/2 tsp/d) [~100%–400% of DRI] or canola oil (~2 tsp/d) [~200% of DRI]	0.5 E% (European Food Safety Authority 2017) [no UL]	ALA‐rich foods should be consumed (Koeder and Perez‐Cueto 2024)	DHA SUPPL should be decided on a case‐by‐case basis. Benefits of DHA SPPL are uncertain. Vegan EPA/DHA supplements based on microalgae oil are available.
	EPA + DHA	No DRI (but DHA is recommended for visual function; 100 mg/d is considered adequate for infants from 6 months) (European Food Safety Authority 2017) [no UL (European Food Safety Authority 2024)]	DHA supply via BM (or formula). If no BM or DHA‐fortified infant formula is consumed, DHA SPPL (50–100 mg/d (Baroni, Goggi, and Battino 2019; European Food Safety Authority 2017)) can be considered.	250 mg/d EPA/DHA + 100–200 mg/d DHA (European Food Safety Authority 2017) [no UL (European Food Safety Authority 2024)]	DHA SPPL in line with the DRI should be considered (Baroni, Goggi, and Battino 2019).	
Selenium (from 4 to 6 months)	15–20 μg/day (European Food Safety Authority 2017; Institute of Medicine 2000) [UL: 4–6 months: 45 μg/day; 7–12 months: 55–60 μg/day (European Food Safety Authority 2024; Institute of Medicine 2000)]	Regions with selenium‐rich soils	No SPPL	70–85 μg/day (55–70 μg/day if not lactating) (European Food Safety Authority 2017; Institute of Medicine 2000) [UL: 255–400 μg/day (European Food Safety Authority 2024; Institute of Medicine 2000)]	No SPPL required	Selenium SUPPL should be decided on a case‐by‐case basis. Excessive selenium intake must be avoided.
		Regions with selenium‐poor soils (Combs 2001)	SPPL should be considered: ~5 μg/day [25%–33% of the DRI] (suggestion: no more than 15 μg/day [75%–100% the DRI])		35–85 μg/day (50%–100% of the DRI)	

Abbreviations: ALA, Alpha‐linolenic acid; BM, Breast milk; BMIC, Breast milk iodine concentration; canola, Rapeseed; DHA, Docosahexaenoic acid; DRI, Dietary reference intake; E%, Percent of energy intake; EPA, Eicosapentaenoic acid; SPPL, Supplementation; tsp., Teaspoon; WHO, World Health Organization.

### 2.2 Vitamin B12

While an amount of 5 μg/day for vitamin B12 supplementation is recommended if one single daily dose is given (Agnoli et al. 2017; Baroni, Goggi, and Battino 2019) (Table 1), the option of 1 μg of vitamin B12 twice per day has also been suggested (Agnoli et al. 2017). The dietary reference intakes (DRIs) for the ages of 4–12 months are somewhat lower, in the range of 0.4–1.5 μg/day (European Food Safety Authority 2017; Institute of Medicine 1998; Ströhle et al. 2019). These DRIs are based on observed intake data in the general (mostly omnivorous) adult population, who typically consume vitamin B12 more than once per day, and have been extrapolated from adults to infants (European Food Safety Authority 2017).

Although vitamin B12 is generally considered nontoxic (Institute of Medicine 1998), adverse effects (Liu et al. 2024), particularly acne‐like skin eruptions, have been reported in some adults, particularly with very high intakes (> 1000 μg/day) (Morales‐Gutierrez et al. 2020). However, anecdotally, this has also been reported with lower intakes. While it is extremely uncertain whether there is a possibility of any serious adverse effects resulting from vitamin B12 supplementation, it appears reasonable to avoid higher doses (e.g., > 10 μg/day) in healthy infants (even though this cutoff is arbitrary, it is > 20 times the DRI in the United States (Institute of Medicine 1998) or > 6 that is in Europe (European Food Safety Authority 2015)). Apart from a study with nonsupplementing macrobiotic vegan (or near‐vegan) women in the United States (1980s) (Specker et al. 1990), only one study to date appears to have reported vitamin B12 concentrations in the breast milk of vegans (Pawlak et al. 2018) (this marker has been reported in vegetarians (Bijur and Desai 1985; Patel and Lovelady 1998; Ureta‐Velasco et al. 2023)). Its results support the assumption that breast milk vitamin B12 levels can be highly variable (Baroni, Goggi, and Battino 2019), even if vitamin B12 is supplemented (Pawlak et al. 2018). Therefore, this study and the lack of other studies appear to justify the recommendation of supplementing vegan infants with vitamin B12 in addition to breast milk (and maternal supplementation) in order to provide additional security (Agnoli et al. 2017; Baroni, Goggi, and Battino 2019). Consequently, there appears to be a consensus that all vegan infants who do not receive infant formula should receive vitamin B12 supplementation. It is urgent to communicate the need for vitamin B12 supplementation to all vegans.

### 2.3 Iodine

Iodine in vegans deserves attention because vegans avoid two main sources of this mineral: Dairy products and fish (Koeder and Perez‐Cueto 2024). Currently, most “plant milks” are not fortified with iodine and therefore lack this nutrient. Only three studies to date appear to have assessed breast milk iodine concentrations (BMIC) among vegans (samples sizes were small). A study from the United States (where iodine levels in iodized salt are relatively high: ~45–50 μg/g (National Health Service 2024)) reported a median BMIC of 62 μg/L in vegans (*n* = 23) and of 93 μg/L in omnivores (*n* = 21) (Pawlak et al. 2023; Perrin et al. 2023). The other two studies did not report BMIC in the vegan group separately from other vegetarians (Spain, vegans: *n* = 11, median BMIC: 121 μg/L [personal communication, NUV [Noelia Ureta‐Velasco]]; Norway, vegans: *n* = 3) (Groufh‐Jacobsen et al. 2020; Ureta‐Velasco et al. 2023).

It should be noted that while a BMIC in the range of 100–200 μg/L (or > 141 μg/L (Pawlak et al. 2023) or ≥ 150 μg/L (Dror and Allen 2018)) is often considered adequate, an optimal BMIC has not been defined, and much higher levels can be observed with a high maternal iodine intake (Andersson and Braegger 2022). The UL for iodine for 1‐year‐old infants is 200 μg/day (Andersson and Braegger 2022; European Food Safety Authority 2006; Institute of Medicine 2001). BMIC appears to increase with maternal iodine intake (Falize et al. 2024) (which is also indicated by the vegans in the Spanish study (Ureta‐Velasco et al. 2023) [personal communication, NUV [Noelia Ureta‐Velasco]]). Therefore, it can be hypothesized that lactating vegan women supplementing 200–290 μg/day (range of DRIs (European Food Safety Authority 2017; Institute of Medicine 2001)) as a single daily dose (Table 1) will likely achieve (or approach) the proposed adequate BMIC of 100–200 μg/L, resulting in an adequate (or near‐adequate) infant iodine intake (70–130 μg/day (European Food Safety Authority 2017; Institute of Medicine 2001)) in those who still consume > 500 mL/d of breast milk. This appears to support the recommendations given (Table 1) (Andersson and Braegger 2022) and would be in line with the recommendation that vegan infants should meet (most of) their iodine requirements through breast milk (or formula) (Baroni, Goggi, and Battino 2019).

If used at all in the infant's food, the seaweed nori (which is sometimes recommended for vegan complementary feeding) should be used in purées (otherwise, it might be a choking hazard). No more than 1/2 sheet of nori (~1 g) per day should be given. On days nori is given, iodine supplements should not be given; nori should not be given every day, and no other seaweed should be given as they can contain excessive amounts of iodine. An adequate supply of iodine via breast milk is optimal, and iodine supplementation should be decided on a case‐by‐case basis.

### 2.4 Vitamin D

Vitamin D supplementation is standard practice for all infants (i.e., independent of diet; Table 1), and there appears to be a consensus that this should also apply to all vegan infants (including those in tropical countries).

### 2.5 Omega‐3 Fatty Acids

As land plants contain virtually no long‐chain omega‐3 fatty acids (eicosapentaenoic acid, EPA; docosahexaenoic acid, DHA), supplementation is frequently recommended, particularly for pregnant and breastfeeding vegans (Baroni, Goggi, and Battino 2019) as DHA is thought to benefit visual function in children and EPA/DHA might contribute to cardiovascular disease prevention (European Food Safety Authority 2017). Particularly, the latter appears uncertain (Sherratt et al. 2024). To date, studies with vegan children regarding visual and cardiovascular function are lacking (Desmond, Fewtrell, and Wells 2024). However, studies with vegan children and anecdotal evidence do so far not provide an indication of impaired visual, cardiovascular, or mental development in vegan children (Sanders 2009). Limited evidence indicates lower circulating cholesterol (total, LDL, and non‐HDL) levels in 2‐ to 18‐year‐old vegans compared to their omnivorous counterparts (Alexy et al. 2021; Desmond et al. 2021; Desmond, Fewtrell, and Wells 2024; Hovinen et al. 2021). Common carotid intima‐media thickness (ccIMT) was nonsignificantly lower (by ~7 μm [~2%]) in vegan compared to omnivorous children (age: ~5–10 years) in Poland (Desmond et al. 2021). This was observed although 56% of the vegan children did not take vitamin B12 supplements, 29% consumed neither vitamin B12 supplements nor vitamin B12‐fortified foods, and 13% had plasma vitamin B12 levels < 200 pg/mL, and vitamin B12 deficiency may be a risk factor for increased ccIMT in vegans (Kwok et al. 2012) (in this Polish study, only 1 of the 52 vegan children took an EPA/DHA supplement [personal communication, MAD [Malgorzata A Desmond]], and EPA/DHA status was not reported) (Desmond et al. 2021). A study from Finland included six vegan children (age: ~2–6 years) and found that they had low serum DHA levels (Hovinen et al. 2021). Similarly, a study from England from about 1990 (long before vegan EPA/DHA supplements became available) included three 14‐week‐old, breastfed, vegan infants and found that they had low levels of DHA in erythrocytes (Sanders and Reddy 1992). Current evidence indicates that EPA/DHA supplementation during pregnancy may reduce the risk of preterm birth and low birth weight (Retterstøl and Rosqvist 2024). However, it is uncertain whether this applies to vegan pregnancies. Limited evidence to date (from Israel and the United States) does not suggest differences between vegan and omnivore pregnancies regarding the risk of preterm birth (Kesary, Avital, and Hiersch 2020) or low birth weight (Avnon et al. 2021; Kesary, Avital, and Hiersch 2020; Pawlak, Ding, and Sovyanhadi 2014). However, EPA/DHA supplementation was not assessed in these studies (Kesary, Avital, and Hiersch 2020; Pawlak, Ding, and Sovyanhadi 2014) or has not yet been published (Avnon et al. 2021) [personal communication YK [Yuval Kesary], RP [Roman Pawlak], TA [Tomer Avnon]]. Benefits of EPA/DHA supplementation for infant health remain uncertain (Retterstøl and Rosqvist 2024). Particularly, preterm infants might benefit (Lundgren et al. 2023), but balancing DHA with arachidonic acid may be needed in this group (Moltu et al. 2024; Rossholt et al. 2023). Consequently, EPA/DHA supplementation of vegan infants should be decided on a case‐by‐case basis (Lemale et al. 2019).

### 2.6 Selenium

Selenium in plant foods is more variable than in animal products. Therefore, increasing selenium intake may be beneficial to vegans in areas with low soil concentrations of this mineral (Koeder and Perez‐Cueto 2024). Selenium has not been highlighted in previous vegetarian complementary feeding guidelines (Agnoli et al. 2017; Baroni, Goggi, and Battino 2019). Only one study to date (United States, where soil selenium levels are relatively high and low selenium status is relatively uncommon (Combs 2001)) appears to have reported selenium concentrations in breast milk of vegans, with a median level of 19 μg/L in vegans (*n* = 23) and of 17 μg/L in omnivores (*n* = 21) (Perrin et al. 2023). Typical breast milk selenium levels in omnivores appear to be in the range of ~10–20 μg/L (Valent et al. 2011; World Health Organization and Food and Agriculture Organization 2004). It can be hypothesized that lactating vegan women in regions with high soil selenium levels (e.g., the United States) or those in regions with low soil selenium who adjust their intake (i.e., moderate supplementation) to achieve the recommended intake range (70–85 μg/day (European Food Safety Authority 2017; Institute of Medicine 2000)) may achieve a similar breast milk selenium level (Perrin et al. 2023). In this regard, the recommendation of a moderate supplementary selenium intake for both mothers and infants (Table 1) appears justifiable for regions with low soil selenium levels. Brazil nuts are sometimes recommended as a selenium source for vegans (Koeder and Perez‐Cueto 2024) but do not appear to be a suitable (main) source of selenium for infants or breastfeeding women. The levels of selenium, barium, and radium (a radioactive element) in these nuts are highly variable and can be excessive (Parekh et al. 2008). Therefore, even Brazil nut butter and ground Brazil nuts should largely be avoided in complementary feeding. Selenium supplementation should be decided on a case‐by‐case basis and should be well below the tolerable upper intake level of 45–60 μg/day (European Food Safety Authority et al. 2023; Institute of Medicine 2000) (Table 1).

### 2.7 Calcium

Calcium is important for bone building. If vitamin D intake is adequate, metabolic calcium deficiency is highly unlikely. As vegans do not consume dairy products, calcium‐rich foods should be consumed (particularly calcium‐fortified foods) by the infant and mother (Koeder and Perez‐Cueto 2024). Calcium‐fortified soya milk (ideally without added sugar or salt) can be used in complementary feeding in small amounts (≤ 250 mL/d) but never as a replacement for breast milk or infant formula (Baroni, Goggi, and Battino 2019). Additional suitable calcium sources include fortified plain yoghurt alternatives, fortified oats, calcium‐set tofu, and pureed, boiled, green leafy vegetables that are rich in calcium and low in oxalic acid (e.g., bok choy, Napa cabbage, broccoli, kale, collard greens, rapini, turnip greens, or mustard greens) (Koeder and Perez‐Cueto 2024). The calcium DRI for 4–6 months is 200 mg/d (Institute of Medicine 2011), and the DRIs for 7–12 months are 260 mg/d (Institute of Medicine 2011) and 280 mg/d (European Food Safety Authority 2017). Low calcium intakes in vegans appear to impair bone health (Tong et al. 2020), although the other micronutrients mentioned in the present article as well as protein are also important in this regard (Koeder and Perez‐Cueto 2024). Home‐based food fortification with calcium (e.g., using *Lithothamnium calcareum* powder or crushed calcium supplements, equivalent to ~100 mg/d) is a possibility and should be decided on a case‐by‐case basis (Lemale et al. 2019).

### 2.8 Iron, Zinc, and Protein

Very limited evidence indicates that vegan children have normal iron stores (Baroni, Goggi, and Battino 2019), and a concomitant intake of vitamin C‐rich foods with iron‐rich plant foods (particularly legumes) increases the presumably lower iron bioavailabilty from plant sources (compared to animal sources) (Baroni, Goggi, and Battino 2019; World Health Organization 2023). Therefore, legumes, together with whole grains and nuts/seeds, appear to constitute a suitable source of iron (as well as zinc and protein), particularly if they are puréed/homogenized (e.g., puréed red lentils, beans, chickpeas; tofu, soya milk; peanut/nut/seed butter (Baroni, Goggi, and Battino 2019)). Nevertheless, iron‐fortified foods (e.g., infant cereal) are frequently recommended (Baroni, Goggi, and Battino 2019), and the European Society for Pediatric Gastroenterology, Hepatology, and Nutrition (ESPGHAN) Committee on Nutrition has stated that all infants should receive iron‐rich complementary foods “including meat products and/or iron‐fortified foods” (Fewtrell et al. 2017). The American Academy of Pediatrics suggests that exclusively breastfed full‐term infants receive iron supplements (1 mg/kg body weight per day) until iron‐rich complementary foods (e.g., iron‐fortified cereals) are introduced (Marra and Bailey 2018). At present, DRIs for infants aged 4–12 months are high (6–11 mg/d) and may not easily be achievable without using iron‐fortified foods (e.g., infant cereal or formula) or iron supplements (Fewtrell et al. 2017). However, excess iron might have pro‐oxidant, pro‐inflammatory effects (Fewtrell et al. 2017; Nchito et al. 2006; Zimmermann et al. 2010), and evidence regarding iron‐fortified foods/supplementation in non‐food‐insecure populations is scarce (World Health Organization 2023). Furthermore, vegan complementary feeding avoids dairy products, which are poor iron sources (low content and bioavailability) and which would otherwise (in non‐vegan feeding) displace iron‐rich complementary foods (e.g., meat or legumes) (Fewtrell et al. 2017). Moreover, delayed umbilical cord clamping can improve infant iron stores and will thus reduce the likelihood of iron supplementation or iron‐fortified foods being required before 6 months (Fewtrell et al. 2017). As such, the necessity of iron‐fortified foods in vegan complementary feeding remains uncertain. Iron or zinc supplementation should be decided on a case‐by‐case basis (Lemale et al. 2019).

### 2.9 Vitamin A

Preformed vitamin A does not occur in plants. Therefore, foods rich in provitamin A (particularly beta‐carotene) should be given, e.g., puréed carrot, orange‐fleshed sweet potato, spinach, mango, papaya, apricots, or red bell peppers. In the United Kingdom, vitamin A (as well as vitamins C and D) supplementation is routinely recommended for infants (from 6 months) who consume ≤ 500 mL/d of infant formula (National Health Service 2024). Vitamin A supplementation is usually not required and should be decided on a case‐by‐case basis.

### 2.10 Suitable Foods

A detailed discussion of suitable foods for vegan complementary feeding is beyond the scope of the present article. In brief, vegan complementary foods can include (all puréed, mashed, or soft boiled) vegetables, tubers (e.g., potatoes without skin), wheat noodles, whole grains (World Health Organization 2023) (e.g., rolled oats), and well‐cooked, well‐puréed legumes (e.g., red lentils, beans, chickpeas), tofu, plain soya milk, plain soya yoghurt, fruit, nut/seed butter (including 100% peanut butter) (Baroni, Goggi, and Battino 2019; World Health Organization 2023), and small amounts of oil (e.g., flaxseed or canola oil) (Baroni, Goggi, and Battino 2019). The World Health Organization recommends a frequent intake of legumes in complementary feeding (World Health Organization 2023). The addition of salt, baking powder/soda, sweeteners (sugar or syrup), and low‐protein milk alternatives (e.g., almond, rice, or oat milk) should be minimized. Higher‐protein milk alternatives that are low in sugar and salt (e.g., plain soya or pea milk) may be used in small amounts (≤ 250 mL/d). Honey (which is not technically vegan (Koeder and Perez‐Cueto 2024)), coffee, tea, and energy drinks should be avoided. Breastfeeding should be continued alongside complementary feeding, at least until 1 year of age but ideally longer, e.g., until the child is 2 years old. If breastfeeding is not possible, the alternatives are breast milk from a human milk bank or soya‐based infant formula (Agnoli et al. 2017) (alternatively rice‐based (Baroni, Goggi, and Battino 2019) or amino acid‐based formula (Kipfer and Goldman 2021)). For more detailed information regarding complementary feeding, the reader is referred to published guidelines (Agnoli et al. 2017; Baroni, Goggi, and Battino 2019; Fewtrell et al. 2017; World Health Organization 2023).

### 2.11 Future Studies and Limitations

Future studies should assess which supplementation regimens (such as the one suggested in Table 1) are ideal for vegan mothers and infants. Adequately powered studies assessing vitamin B12, iodine, vitamin D, and selenium in spot breast milk samples of vegan women should therefore be conducted (Andersson and Braegger 2022). Ideally, repeated milk samples as well as detailed information regarding supplement intake should be obtained (Ureta‐Velasco et al. 2023). Furthermore, the potential benefits of iron‐ and/or zinc‐fortified foods for vegan infants should be explored. More research is also needed on whether some vegan infants may benefit from the supplementation of other nutrients, e.g., choline (European Food Safety Authority 2016), arachidonic acid (Moltu et al. 2024), or taurine. Randomized controlled trials in which infants or families would be assigned to a well‐planned vegan or omnivorous diet are difficult to conduct, both for ethical (due to the lack of evidence) and practical (due to the lack of willing participants) reasons. In addition, transdisciplinary nutrition education for vegans is needed. A desirable scenario would be a cooperation of scientists and pro‐vegan organizations providing adequate guidance to vegan parents. While current evidence does not provide answers to all questions, decades of experience as well as some studies with vegan children indicate clear requirements in some areas, first of all vitamin B12 supplementation in all vegan children. As the lack of vitamin B12 supplementation in the abovementioned study from Poland shows, even well‐established requirements are frequently still not put into practice (Desmond et al. 2021). It is well established that diets that lack vitamin B12 (or any of the other abovementioned nutrients) will lead to impaired health. Therefore, it can be proposed that studies with vegans whose intake is deficient will not provide new knowledge, apart from the observation that a considerable percentage of vegans is still not educated in this regard. In contrast, a combination of vegan nutrition education and the assessment of nutrient and health status will generate new knowledge in terms of how vegan diets can be made safer for those who choose this way of eating (often with very strongly held convictions). While observational studies with vegan families assessing dietary intake and nutrient status are also required, without nutrition education, vegans are left to their own devices to find the right dietary strategies.

However, beyond the scope of the present article, it is worth noting that nutrition education does not equate to adequate application. The formulation of guidelines is fundamental to nutrition education, which in turn is essential for applied nutrition knowledge and its health effects. Future research will be needed to assess whether vegans with adequate nutrition knowledge can effectively apply this knowledge.

Dietary reference intakes vary internationally, and this lack of consensus is more obvious for certain micronutrients. For example, the adequate intake for vitamin B12 for infants aged ≥ 6 months is 0.5 μg/day in Japan and the United States (Institute of Medicine 1998; Ministry of Health, Labour and Welfare 2019) but 1.5 μg/day in France (ANSES 2021) (and many other European countries). Similarly, recommended intakes for selenium for this age group range from ~10 μg/day in the Philippines (Food and Nutrition Research Institute 2018) to 20 μg/day in the United States (Institute of Medicine 2000). However, while future research may result in greater consensus, these ranges can already provide guidance today.

The bioavailability of the key nutrients discussed above can be assumed to be relatively high from supplements and fortified foods, irrespective of the different vitamers (e.g., cyanocobalamin vs. methylcobalamin (Obeid, Fedosov, and Nexo 2015)) or mineral species (e.g., sodium selenate/selenite vs. selenomethionine (Burk and Hill 2015)) and irrespective of whether they are taken with or in between meals (Garrod et al. 2019). Therefore, such further differentiation can be considered of secondary importance.

## Author Contributions

**Christian Koeder:** conceptualization (lead), writing – original draft (lead), writing – review and editing (lead).

## Conflicts of Interest

The author declares no conflicts of interest.

## Data Availability

The author has nothing to report.
